# On the biological activity of cytokinin free bases and their ribosides

**DOI:** 10.1007/s00425-021-03810-1

**Published:** 2021-12-23

**Authors:** Georgy A. Romanov, Thomas Schmülling

**Affiliations:** 1 Timiryazev Institute of Plant Physiology, Russian Academy of Sciences, Botanicheskaya 35, 127276 Moscow, Russia; 2 Institute of Biology/Applied Genetics, Dahlem Center of Plant Sciences, Freie Universität Berlin, Albrecht-Thaer-Weg 6, 14195 Berlin, Germany

**Keywords:** 3D protein structure, Cytokinin receptor, Cytokinin riboside, Cytokinin signaling, Plant hormone, Structure–activity relation

## Abstract

**Main conclusion:**

The free bases of cytokinins are the biologically active forms of the hormone while cytokinin ribosides become active only upon removal of the ribose residue.

**Abstract:**

Cytokinins (CKs) belong to the classical plant hormones. They were discovered more than 65 years ago, but which molecular forms possess genuine CK activity is still matter of debate. Numerous studies support the view that only the free bases are the biologically active molecules. This standpoint has been challenged in a recent review (Nguyen et al. in Planta 254: 45, 2021) proposing that also CK ribosides may have genuine own CK activity. Here we critically discuss the pros and cons of this viewpoint considering the results of biological assays, CK binding studies, 3D structural data of CK**-**receptor interaction and mutant analyses. It is concluded that all types of study provide clear and convincing evidence only for biological activity of free bases and not ribosides; the latter are rather a transport form of the hormone without their own biological activity.

## Introduction

Intrinsic hormonal activity of a compound implies its ability to affect the receptor directly, switching it from one state to another. It is important to distinguish the biologically active compound of a given hormone from related compounds, which might be metabolic intermediates, storage or transport forms of the hormone. This is also true for cytokinins (CKs), which belong to the classical plant hormones with a broad spectrum of activity *in planta*. Chemically they are adenine derivatives carrying *N*^6^-linked side chain. The main forms are isopentenyladenine (iP), *trans*- and *cis*-zeatin (tZ and cZ). They typically occur as a mixture in plants, including free bases (or nucleobases), their ribosides and ribotides, as well as *O*-, *N*7- and *N*9-linked sugar conjugates. Recently, a review appeared in Planta focusing on CK ribosides and describing their history, metabolism, transport and putative functions (Nguyen et al. 2021). Importantly, the authors propose that the long-standing view that the active CKs are their free base forms should be expanded to include respective ribosides (Nguyen et al. 2021). The main arguments put forward by the authors has been the correlation of high concentrations of CK ribosides with CK activities *in planta* and their apparent ability to bind to CK receptors in certain heterologous test systems mentioned further below. The question whether or not CK ribosides are active CKs has been matter of debate during the history of CK studies. Therefore, this question and the arguments put forward by Nguyen et al. (2021) in favor of a genuine CK activity of CK ribosides need to be considered carefully. In this commentary, we are going to describe the current knowledge and give an oversight of the pros and cons. It will be concluded that most if not all of the available data do not support genuine CK activity of the ribosides.

### Historical outline

CKs were first discovered in the form of kinetin, an artificial *N*^6^-modified nucleobase (Miller et al. 1955). Almost 10 years later the first natural CK–nucleobase termed zeatin was isolated from *Zea mays* seeds (Letham 1963). Afterwards various additional CKs and their derivatives were discovered as free molecules and constituents of tRNA, among them CK nucleosides with a ribose residue at the *N*9 of the adenine heterocycle (Skoog and Armstrong 1970). Because CK ribosides are often abundant and display CK activity in many bioassays, the question arose whether they possess genuine CK activity. Already at that time there were indications that they lack own CK activity. Addition of a non-hydrolyzable ribose group to *N*9 of a CK analog practically eliminated its CK activity (Hecht et al. 1971). It was also demonstrated that typical *N*9-substituted CKs can rapidly (within a few minutes of incubation) loose the *N*9-substitution in contact with living tissue (Fox et al. 1971). Unlike CK bases, the ribosides had no significant CK activity in the *Funaria* biotest (Spiess 1975). All this led to the widespread viewpoint that ribosides have no own activity (Sakakibara 2006).

### Ligand binding to CK receptors

The question which CK compounds have biological activity emerged again after the discovery of CK receptors (Inoue et al. 2001; Suzuki et al. 2001). The first studies were limited to the three Arabidopsis CK receptors, termed AHK2, AHK3 and CRE1/AHK4. Checking the binding activities of free bases and ribosides using transgenic *E. coli* expressing the *CRE1/AHK4* receptor gene yielded positive results for both compounds (Yamada et al. 2001). At the same time, evaluation of the ligand binding capabilities of the CRE1/AHK4 receptor residing in membranes isolated from yeast expressing the *CRE1*/*AHK4* gene revealed the inability of ribosides, exemplified by iPR, to bind to the receptor (Yamada et al. 2001). However, subsequently the *E. coli* test system was developed as a convenient tool for quantitative analysis of hormone-receptor interactions (Romanov et al. 2005), and in this (Romanov et al. 2005; Stolz et al. 2011; Kuderová et al. 2015) and similar (Spíchal et al. 2004) test systems the CK ribosides usually exhibited binding/activity with rather high affinity for different CK receptors. Therefore, at that time many researchers believed that ribosides might possess their own CK activity.

### CK receptor 3D structure

Ten years ago, Hothorn et al. (2011) succeeded in crystallizing the sensory module of the CRE1/AHK4 receptor, which allowed to readdress the question of “true ligands” with a novel approach. Analysis of the 3D structure of the binding site showed a space deficiency to accommodate the ribose residue in the correct nucleobase orientation. Hothorn et al. (2011) stated: "Although the structure strongly suggests that *N*3-, *N*7-, and *N*9-substituted cytokinins should not bind effectively to AHK4, previous studies using hormone binding assays in live *E. coli* cells have shown that, for example, *trans*-zeatin riboside (tZR) *N*9-substituted cytokinin binds to AHK4 with high affinity. To resolve this discrepancy, we purified AHK4 in the presence of tZR and crystallized the complex. However, we cannot detect any density differences for the ribose portion of tZR, suggesting that the ligand may have undergone hydrolysis during purification, a reaction that can also occur in hormone binding assays in living cells." Modeling of the 3D structures of the sensory modules of CK receptors from different species (Arabidopsis, maize, tobacco, potato, rice) pointed to their high structural conservation and especially of their binding sites, adjusted to bind specifically CK nucleobases and not ribosides (Lomin et al. 2015, 2018b; Savelieva et al. 2018; Arkhipov et al. 2019; and data not published). Notably, also iP9G and tZ9G, which carry glucosides at the *N*9 position of iP and tZ, did not inhibit binding of the respective free bases on AHK3 and CRE1/AHK4 receptors (Šmehilová et al. 2016). This result supports the viewpoint that attachment of groups at the *N*9 position interferes with receptor binding.

### Plant membrane assay system

**Fig. 1:**
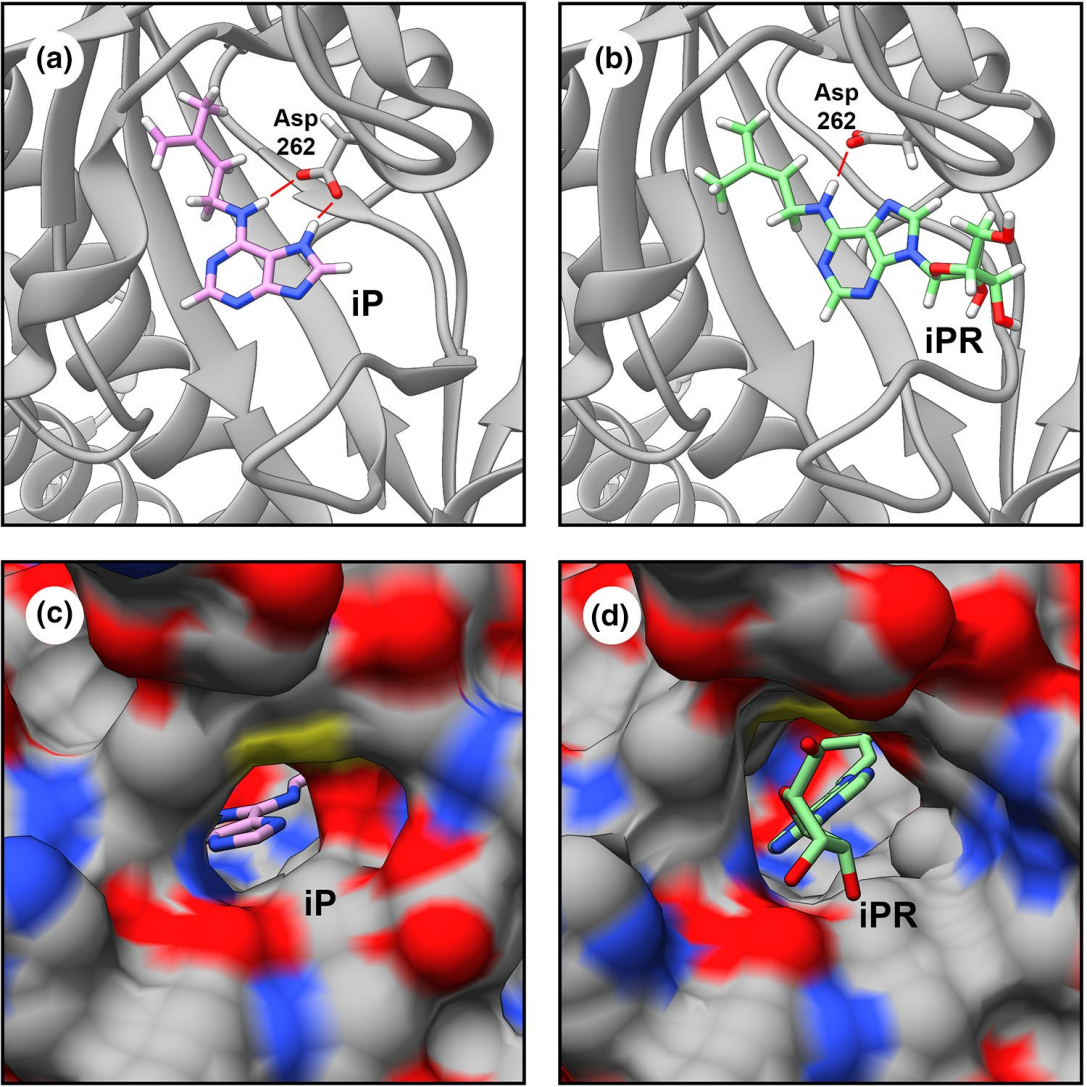
Schematic presentation of the CK binding site in the sensory module of the CRE1/AHK4 receptor (AHK4sm) of *A. thaliana*. **a** and **c** Crystal structure of the AHK4sm–iP complex (PDB ID: 3T4J) (Hothorn et al. 2011). **b** and **d** Protein–ligand docking of the iPR molecule into AHK4sm, performed using the Rosetta software (Rosie web service) (DeLuca et al. 2015). **a** and **b** 3D presentations showing the binding of iP and iPR to the ligand binding site of CRE1/AHK4. Numbering of the Asp residue is according to the crystal structure of CRE1/AHK4 (Hothorn et al. 2011). **c** and **d** Comparison of iP and iPR positions in the ligand-binding pocket of CRE1/AHK4sm. The protruding "tail" of the ribose of iPR does not fit inside the ligand-binding pocket. Visualization was performed using UCSF Chimera software (Pettersen et al. 2004)

The apparent conflict between the results obtained in the *E. coli* test system and those obtained by other methods was resolved by Lomin et al. (2015). They described a new method for quantitative analysis of the CK receptor–ligand interaction in microsomes isolated from tobacco leaves transiently expressing one or the other individual CK receptor. In comparative experiments, it was found that the apparent activity of CK ribosides, in contrast to nucleobases, is strongly dependent on the test system. In the *E. coli* system, ribosides repeatedly showed relatively high affinity to the receptors, but in the system with plant microsomes, the apparent affinity of ribosides for receptors (*K*_a_) dropped by orders of magnitude. The binding of CK ribosides to receptors in the plant test system was in most cases so small that the ligand–receptor affinity (*K*_a_ or *K*_d_) could be not be determined (Lomin et al. 2015). In subsequent experiments using the plant test system, it turned out that not only addition of ribose but also of other substituents at the *N*9-position of the adenine heterocycle dramatically decreases the ligand affinity to receptors, turning biologically active adenine derivatives into inactive compounds (Savelieva et al. 2018). A detailed structural analysis showed that due to *N*9 substitution, a H-bond donor cannot emerge at *N*7, this H-bond with Asp262 in the ligand-binding domain playing a key role in the ligand-specific recognition (Fig. 1). Moreover, hydrogen bonding of CK ribosides with Leu194 is realized through *N*3 instead of *N*9, leading to the elimination of a favorable interaction with a water molecule. Furthermore, a ribose residue in the ligand molecule prevents the closure of the cavity by the β6-β7 loop (Fig. 1). Without site closure, specific high-affinity ligand binding by the CK receptor leading to receptor activation is hardly possible (Lomin et al. 2015).

### Receptor binding vs. receptor activation

Unfortunately, the commented review (Nguyen et al. 2021) considers neither the information obtained by 3D modeling of the receptor ligand-binding domain nor the results from binding studies performed with plant membranes. The opinion put forward was based mainly on the correlation of high concentrations of CK ribosides with biological activity and binding studies performed with the *E. coli* test system, including data of Romanov et al. (2006), Stolz et al. (2011) and Kuderová et al. (2015). But as it was mentioned above, the *E. coli* assay was shown to be not appropriate for CK riboside investigation. A further work by Daudu et al. (2017), which was cited to prove CK riboside activity but not discussed, assessed semiquantitatively the ligand preference of CK receptors of apple tree using a yeast test system. It was concluded that of the five tested CK receptors of apple, four do not respond to CK ribosides at all. Only one receptor, MaCHK2, was apparently able to react when exposed to CK ribosides, but its activation by ribosides required 500–1000 fold higher concentrations compared to the cognate tZ– and iP–nucleobases (Daudu et al. 2017). Taking into account that some tiny contamination (~ 0.1%) of the ribosides with cognate nucleobases can be hardly ruled out, it is doubtful that this particular apple tree receptor recognizes CK ribosides as activating ligands *in planta*. Another recent article by Jaworek et al. (2020) showing that CK ribosides are able to bind with high affinity to poplar CK receptors was not mentioned by Nguyen et al. either. In this article, the conversion of iPR to iP during the bacterial binding assay was indeed recorded but found to be low, which argued for relatively strong binding of iPR themselves to poplar CK receptors. To explain this result, Jaworek et al. (2020) suggested an induced fit, rearrangement of the loop close to the nucleobase or eventually a nucleobase flip that may occur *in planta* leading to CK riboside binding by the receptor. However, despite this binding, the activation of the poplar CK receptors by iPR was not reported (Jaworek et al. 2020). Indeed, high affinity binding of a compound to the receptor is not necessarily associated with the induction of hormonal activity. In a study of numerous CK derivatives recognized by CK receptors, inert ones or even compounds with opposite (inhibitory) action were quite common (Savelieva et al. 2018).

### *N*9-bound fluorescent probe of CK

Lack of activation of a receptor by a binding compound was also found in a study using a fluorescently labeled CK that interacts with the receptor CK binding site. This fluorescent probe contains 7-nitro-2,1,3-benzoxadiazole (NBD), a small fluorophore comparable in size to a ribose residue and attached to the *N*9 position of iP (Kubiasová et al. 2020). Docking simulations using the CRE1/AHK4–iP crystal structure suggested that iP-NBD may be fully embedded into the active sites of AHK receptors. Competitive binding assays with *E. coli* expressing either AHK3 or CRE1/AHK4 showed that iP-NBD competes for receptor binding with radiolabelled *trans*-zeatin (tZ), with *K*_i_ ~ 37 μM in AHK3 and *K*_i_ ~ 1.4 μM in CRE1/AHK4 assays. This means that the attached *N*9 residue decreased the affinity of modified iP to CK receptors by about three to four orders of magnitude. Despite iP-NBD fitted into the CK-binding pockets of the receptors, it failed to trigger a CK response in an *E. coli* receptor activation assay with recombinant AHK3 and CRE1/AHK4 receptors, and it failed as well to induce the CK reporter genes *TCS::GFP* and *ARR5::GUS in planta* (Kubiasová et al. 2020). This strongly suggested that similar to CK ribosides and other *N*9 derivatives, iP-NBD binds to CK receptors but does not activate them, thus working as a partial CK receptor antagonist. Consistent with its highest affinity to CRE1/AHK4, iP-NBD had a pronounced inhibitory effect on the induction of a CK response gene (*ARR5*) in the *ahk2ahk3* double mutant with only the CRE1/AHK4 receptor functional when compared to either wild type or the other CK receptor double mutants (*ahk3ahk4* and *ahk2ahk4*) (Kubiasová et al. 2020).

### CK mutants

Additional results supporting CK activity of the free bases but not of the ribosides come from the analysis of CK mutants of Arabidopsis, however these publications were not considered either by Nguyen et al. (2021). In the *LOG* sextuple Arabidopsis mutants, which are deficient in the production of iP and tZ from the nucleotides iPRP and tZRP, the iPR and tZR content was about six- and threefold higher compared to wild-type plants. However, this excess of iPR and tZR could not compensate for a much lower deficiency in iP and tZ bases (Tokunaga et al. 2012). Furthermore, mutant analyses and grafting experiments have shown that the activity of root-derived tZR depends on its metabolic conversion to the free base through the LONELY GUY(LOG)-mediated pathway (Osugi et al. 2017), possibly involving an adenosine kinase activity as a first step to form intracellular ZRMP as an intermediate compound (Lomin et al. 2018a; Romanov et al. 2018; Sakakibara 2021).

## Concluding remarks

In summary, structural data, receptor-ligand interaction studies and mutant analyses argue, in our opinion quite convincingly, against a genuine biological activity of CK ribosides. The hormonal activity of CK ribosides found in experimental systems is most likely due to a rapid hydrolytic removal of the ribose moiety thus releasing the active CK base. The prevailing viewpoint that CK nucleobases are the only active forms is documented in more recent fundamental reviews (Kieber and Schaller 2018; Oschepkov et al. 2020). Nevertheless, despite the lack of own CK activity the ribosides are an important cellular source of active CK corresponding with their high concentrations in tissues with high CK activity. Therefore, it would be important to explore the metabolism of CK ribosides and their role as a transport form, as well as active CK precursors, in more detail. In particular, identification of the genes coding for specific enzymes catalyzing the interconversion between CK free bases and ribosides will be of great value and open up new avenues of research.

### *Author contribution statement*

GAR and TS designed the project, GAR wrote the draft, TS and GAR prepared the final version.

## Abbreviations

CK: Cytokinin
cZR: *cis*-Zeatin riboside
iP: Isopentenyladenine
iPR: Isopentenyladenine riboside
tZ: *trans*-Zeatin
tZR: *trans*-Zeatin riboside
